# The value of 3D contrast-enhanced CT radiomics in predicting response to neoadjuvant chemotherapy for adenocarcinoma of the esophagogastric junction: a two-center study

**DOI:** 10.1186/s12885-026-15609-y

**Published:** 2026-01-23

**Authors:** Chenglong Luo, Jing Li, Wanling Mu, Mengchen Yuan, Pengchao Zhan, Yiyang Liu, Yue Zhou, Liming Li, Changmao Ding, Xuejun Chen, Jianbo Gao

**Affiliations:** 1 Department of Radiology, The First Affiliated Hospital of Zhengzhou University, Zhengzhou, Henan province 450052 China; 2 Henan Key Laboratory of Imaging Diagnosis and Treatment for Digestive System Tumor, Zhengzhou, Henan province 450052 China; 3 Department of Radiology, The Affiliated Cancer Hospital of Zhengzhou University, Zhengzhou, Henan province 450008 China

**Keywords:** Computed tomography, Esophagogastric junction, Adenocarcinoma, Neoadjuvant therapy, Response evaluation, Nomogram

## Abstract

**Background:**

To investigate the feasibility of 3D contrast-enhanced CT radiomics features to predict response to neoadjuvant chemotherapy (NAC) for adenocarcinoma of the esophagogastric junction (AEG) and to develop and validate a nomogram to assist in clinical decision-making.

**Methods:**

The clinical, pathological, and CT data of 239 patients with locally advanced AEG who underwent NAC and radical resection were retrospectively collected between March 2016 and June 2023 from two independent Chinese medical centers. They were randomly assigned to a training cohort, an internal verification cohort, or an external verification cohort. Based on the CT radiomics features after dimension reduction, the radiomics model was constructed using linear discriminant analysis as the classifier to obtain the radiomics score. Clinical characteristics were screened, and multivariable logistic regression was applied to construct the clinical model. The combined model was generated by integrating clinical features and radiomics scores, upon which a nomogram was subsequently developed. Finally, receiver operating characteristic curves, calibration curves, and decision curves were plotted to evaluate the predictive performance, calibration performance, and clinical benefits of each model for the efficacy of NAC in AEG patients.

**Results:**

Overall, 86 of the 239 patients responded well to NAC. The nomogram was comprised of tumor thickness, lymph node short diameter, and the radiomics score. In the training cohort, the AUC values of the clinical model, the radiomics model, and the combined model for predicting NAC response were 0.771 (95% CI, 0.682–0.860), 0.823 (95% CI, 0.742–0.903), and 0.894 (95% CI, 0.834–0.954), respectively, with the combined model displaying optimal discriminatory power. The combined model also demonstrated satisfactory predictive performance in the internal and external validation cohorts, with AUC values of 0.859 and 0.775, respectively. The calibration curves for the three cohorts showed good agreement between predictions and actual observations. Lastly, decision curve analysis highlighted the clinical applicability of the combined model.

**Conclusion:**

The nomogram integrating radiomics and clinical characteristics demonstrated good performance in predicting NAC response in AEG, suggesting its possible role as a decision-support tool for treatment individualization. These preliminary findings warrant confirmation in future studies.

**Supplementary Information:**

The online version contains supplementary material available at 10.1186/s12885-026-15609-y.

## Background

Although the incidence of gastric cancer has progressively decreased over the past 40 years, the morbidity and mortality rates of adenocarcinoma of the esophagogastric junction (AEG) remain high [1–3]. An analysis of global gastric cancer and esophageal cancer registration data in 2018 revealed that new cardiac cancers in East Asia accounted for 67.1% of the world’s total, a significant increase compared with 2012 data [2, 4]. Notably, the overall prognosis of AEG is poorer than distal gastric cancer due to its unique biological characteristics. Siewert et al. [5] reported that in Western countries, over 80% of AEGs are progressive, with a 5-year overall survival rate below 30%. The results of previous studies have concluded that compared with surgery alone, neoadjuvant chemotherapy (NAC) for locally advanced esophagogastric junction cancer can aid in achieving local tumor control and prolonging patient survival [6–8]. Thus, the combination of NAC with surgical resection has gradually emerged as the standard treatment for locally advanced AEG [9]. However, not all AEG patients benefit from NAC due to tumor heterogeneity [10, 11]. Some patients respond poorly to NAC and experience toxic side effects, leading to delayed surgery, tumor progression, and poor prognosis [8, 12]. Therefore, there is a pressing need to predict the pathological response of AEG to NAC.

With advances in AEG comprehensive treatments and the promotion of multidisciplinary diagnosis and treatment modalities, the demand for individualized diagnosis and treatment for accurate imaging evaluation is increasing. Multi-slice spiral CT examination is currently one of the main approaches for baseline examination prior to NAC and post-treatment evaluation of AEG. However, due to the special anatomical location of AEG, the response evaluation criteria in solid tumors do not adequately meet clinical needs. Radiomics, as a non-invasive image analysis method, extracts and integrates the massive information contained in tumor images, from basic morphology to texture features to complex high-dimensional features, and subsequently constructs a decision model to assist in clinical evaluation through dimension reduction and modeling [13]. Earlier studies have identified that some radiomics features are significantly related to chemotherapy response, and models based on radiomics features show potential and advantages in baseline prediction of NAC efficacy in digestive tract cancer [14–16].

Early identification of the therapeutic response of AEG to NAC is key for limiting chemotherapy toxicity and guiding individualized treatment strategies. However, relevant radiomics studies specifically targeting AEG are scarce. Therefore, this study aimed to summarize the clinical and pathological characteristics of locally advanced AEG, integrate 3D contrast-enhanced CT radiomics features to construct a model and analyze and evaluate the predictive value of the model for NAC response.

## Materials and methods

The whole study was performed by the World Medical Association guidelines and Declaration of Helsinki, revised in 2024. This retrospective study was approved by the Institutional Review Board of the the First Affiliated Hospital of Zhengzhou University, and the requirement for informed consent was waived.

### Patient enrollment

**Fig. 1:**
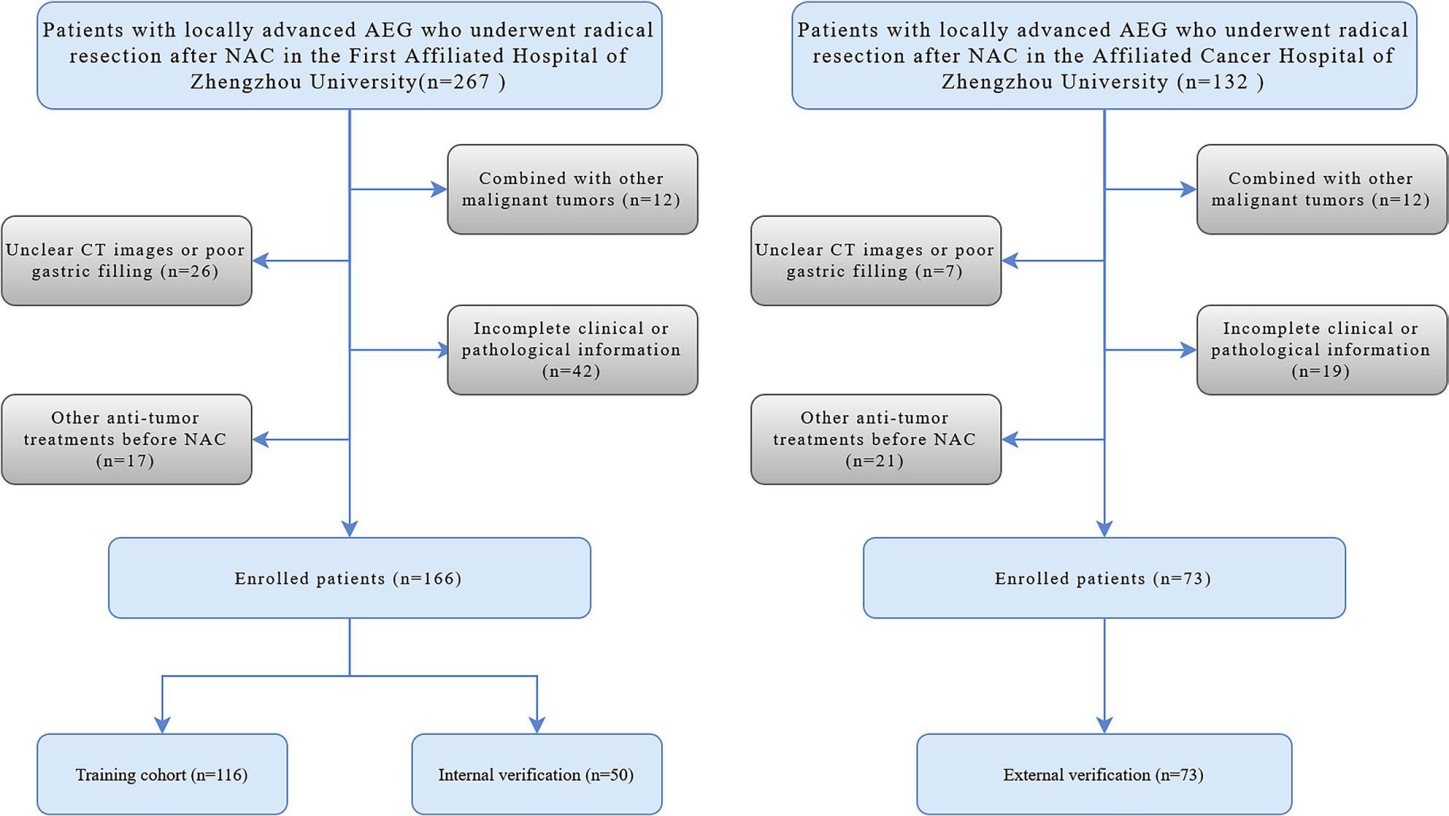
Patient flowchart for this study

The clinical information of patients with locally advanced AEG who underwent NAC and radical surgical resection in the First Affiliated Hospital of Zhengzhou University (center 1) and the Affiliated Cancer Hospital of Zhengzhou University (center 2) from March 2016 to June 2023 were retrospectively collected. The inclusion criteria were as follows: (a) Histopathologic confirmation of locally advanced AEG (clinical stage T2-4aNxM0) prior to NAC initiation. (b) Contrast-enhanced CT performed within one week prior to initiating NAC. (c) Receipt of 2–4 cycles of NAC before surgical resection. (d) Radical resection performed after NAC. The exclusion criteria were as follows: (a) Presence of other malignant tumors. (b) Unclear CT images or poor gastric filling. (c) Other anti-tumor treatments before NAC. (d) Incomplete clinical or pathological information. The patient inclusion process is illustrated in Fig. 1. A total of 166 patients with AEG from the center 1 were randomly divided into a training cohort (*n* = 116) and an internal verification cohort (*n* = 50) in a 7:3 ratio, while 73 AEG patients from the center 2 were included in the external verification cohort (*n* = 73).

### Clinical and pathological data

This retrospective study analyzed clinical and pathological data from 239 patients with AEG. Collected variables included age, sex, smoking, alcohol drinking, Siewert classification, Lauren classification, tumor differentiation grade, clinical TNM stage, serum levels of carcinoembryonic antigen (CEA; normal: 0–5 ng/mL), carbohydrate antigen 199 (CA199; normal: 0.01–27 U/mL), and carbohydrate antigen 125 (CA125; normal: 0.01–35 U/mL). Additional data comprised NAC regimen, total NAC cycles, tumor morphological features (necrosis, serosal invasion, tumor thickness, longest diameter, surface area, volume, and surface area/volume), and lymph node short diameter. Clinical TNM stages were assessed according to the 8th edition of the American Joint Committee on Cancer (AJCC) criteria. Tumor morphological parameters were measured on the 3D Slicer (version 5.6.2; https://www.slicer.org/) software, wherein the tumor thickness and longest diameter were defined as the maximum values measured at the two-dimensional level, and the tumor surface area and volume were evaluated and calculated at the three-dimensional level. Lymph node stations 1–12, 19–20, and 110–111 were assessed. Lymph nodes with a short diameter ≥ 8 mm were considered measurable [17]. The lymph node short diameter was calculated as the sum of the short diameters of the five largest measurable lymph nodes, or of all measurable nodes in cases with fewer than five. All CT-based qualitative and quantitative parameters were initially assessed by a junior radiologist (W.L.M.), and then reviewed and corrected by a senior radiologist (Y.Z.). Any discrepancies were resolved through discussion to reach a final consensus, thereby ensuring the reliability of the measurements.

NAC regimens included oxaliplatin + S-1 (SOX), capecitabine + oxaliplatin (XELOX), and 5-fluorouracil + leucovorin + oxaliplatin + docetaxel (FLOT). Tumor regression was evaluated in postoperative pathological specimens according to the Becker grading system [18]. Briefly, grade 1a was defined as the absence of residual tumor cells; grade 1b was defined as < 10% residual tumor cells; grade 2 was defined as 10–50% residual tumor cells; and grade 3 was defined as > 50% residual tumor cells. According to the grading results, the response to NAC was categorized into two groups: grades 1a and 1b were classified as the good response (GR) group, whereas grades 2 and 3 were classified as the poor response (PR) group [19].

### CT image acquisition

The CT image acquisition parameters are provided in Supplementary A1.

### Tumor segmentation and radiomics feature extraction

Venous phase images of AEG patients with a layer thickness of 5 mm were uploaded to the Deepwise Multimodal Research Platform (version 2.5.1; https://keyan.deepwise.com, Hangzhou Deepwise & League of PHD Tecfvhnology Co., Ltd, Hangzhou, Zhejiang, China.). The specific operation details and tumor segmentation examples are shown in Supplementary A2 and Figure S1, respectively.

### Radiomics feature selection

Z-normalized scaling of the extracted features was performed using the following formula: z = (x - mean)/std. Intraclass correlation coefficient analysis (ICC) was carried out, and radiomics features with ICC < 0.80 were excluded to guarantee the selection of features with high repeatability for the ensuing analyses. Next, feature correlation analysis was performed, and the threshold of the correlation coefficient was set to 0.65 to minimize the redundancy between features. Finally, the F-test was used for feature selection to retain features with significant differences between groups.

### Model construction and evaluation

In the training cohort, multivariable logistic regression was performed on clinical characteristics with statistically significant differences to construct the clinical model. Based on the screened radiomics features, linear discriminant analysis (LDA) was used as the classifier to construct the radiomics model, and the radiomics score was calculated. The combined model was generated using multivariable logistic regression by incorporating key clinical characteristics and radiomics scores, which was then used to develop a nomogram. Receiver operating characteristic (ROC) curves were plotted to analyze the efficacy of each model in predicting NAC response, which was evaluated in the internal and external verification cohorts. The Hosmer-Lemeshow test was used to evaluate the goodness of fit of the model, with *P* > 0.05 considered a good model fit. The area under the curve (AUC) of each model was compared using the DeLong test. Calibration curves were drawn to evaluate the calibration performance of each model. Decision curve analysis (DCA) was conducted to evaluate the clinical usefulness of the models.

### Statistical analysis

Statistical analysis were performed using SPSS 25.0 and Medcalc 20.015 statistical analysis software. Continuous variables that conformed to the normal distribution were expressed as mean ± standard deviation and compared using the independent sample t-test; continuous variables that were not normally distributed were expressed as the median (interquartile range) and compared using the Mann-Whitney U test. Categorical variables were compared using the χ² test or Fisher exact test. R software (version 4.4.0) was used for model construction and performance evaluation. A two-tailed p-value of < 0.05 was considered statistically significant.

## Results

### Clinical characteristics

A total of 239 patients with locally advanced AEG were included in this study according to inclusion and exclusion criteria, of whom 86 (36.0%) demonstrated a favorable response to NAC. The clinical characteristics of all patients are summarized in Table 1. In the training cohort, significant differences were noted in tumor thickness and lymph node short diameter between the PR and GR groups (*P* < 0.05), whereas the remaining clinical characteristics were comparable between the groups (*P* > 0.05). The results of multivariable logistic regression analysis identified tumor thickness (odds ratio [OR] 0.155, 95% confidence interval [CI] 0.052–0.460, *P* = 0.001) and lymph node short diameter (OR 0.754, 95% CI 0.595–0.955, *p* = 0.019) as independent predictors of the response to NAC for AEG patients, and the clinical model was constructed accordingly.

**Table 1:** Baseline characteristics of patients with adenocarcinoma of esophagogastric junction

Characteristic	Training cohort (*n* = 116)			Internal verification (*n* = 50)			External verification (*n* = 73)		
	GR(*n* = 40)	PR(*n* = 76)	*p* value	GR(*n* = 17)	PR(*n* = 33)	*p* value	GR(*n* = 29)	PR(*n* = 44)	*p* value
Age (years)^a^	61.730± 7.222	62.36± 8.885	0.700	61.590± 9.166	62.270± 6.573	0.762	63.241± 9.609	64.364± 8.678	0.606
Sex			1.000			0.047			0.919
Men	37	69		10	29		26	41	
Women	3	7		7	4		3	3	
Smoking			0.253			0.623			0.669
No	27	43		12	21		15	25	
Yes	13	33		5	12		14	19	
Alcohol drinking			0.756			1.000			0.786
No	29	53		13	25		20	29	
Yes	11	23		4	8		9	15	
Siewert classification			1.000			0.839			0.566
I	5	9		1	2		2	3	
II	27	52		15	27		18	22	
III	8	15		1	4		9	19	
Lauren			0.233			0.111			0.337
Intestinal type	25	35		13	17		10	15	
Mixed type	10	29		2	13		7	17	
Diffuse type	5	12		2	3		12	12	
Differentiation			0.163			0.020			0.737
Low	21	50		6	23		18	29	
Middle-high	19	26		11	10		11	15	
Clinical TNM stage			0.186			0.084			0.709
2	1	1		0	0		0	1	
3	15	42		7	22		16	23	
4a	24	33		10	11		13	20	
CEA (Elevated)	13	28	0.642	9	9	0.073	7	14	0.478
CA199 (Elevated)	8	19	0.545	3	7	1.000	6	13	0.399
CA125 (Elevated)	7	6	0.212	1	1	1.000	1	1	1.000
NAC regimen			0.431			0.376			0.795
SOX	32	54		12	28		20	33	
FLOT	3	12		4	3		5	7	
XELOX	5	10		1	2		4	4	
Total cycles of NAC			0.369			0.537			0.963
2	18	36		6	17		10	16	
3	15	20		8	11		13	20	
4	7	20		3	5		6	8	
Necrosis			0.303			0.242			0.120
Absent	31	52		9	23		26	33	
Present	9	24		8	10		3	11	
Serosal invasion			0.461			0.616			0.455
Absent	28	48		8	18		18	31	
Present	12	28		9	15		11	13	
Lymph node short diameter(cm) ^b^	1.690 (2.985)	2.710 (3.075)	0.007	1.760 (3.210)	2.781 (2.020)	0.035	1.380 (2.020)	2.316 (3.062)	0.008
Tumor thickness(cm) ^b^	1.486 (0.379)	1.773 (0.672)	<0.001	1.421 (0.776)	1.712 (0.436)	0.034	1.365 (0.275)	1.679 (0.467)	0.004
Longest diameter(cm) ^b^	5.919 (2.853)	5.968 (3.49)	0.889	6.261 (1.431)	6.117 (2.179)	0.846	7.494 (2.882)	7.141 (2.448)	0.861
Surface area(cm^2^) ^b^	71.095 (34.186)	72.901 (28.158)	0.866	76.754 (32.970)	70.009 (30.611)	0.690	77.610 (54.610)	78.948 (39.239)	0.973
Volume(cm^3^) ^b^	22.958 (13.701)	23.628 (13.783)	0.634	26.667 (12.911)	22.598 (15.256)	0.395	20.171 (14.606)	24.457 (12.565)	0.478
Surface area/volume ^b^	3.193 (0.861)	3.025 (0.921)	0.371	2.845 (0.745)	3.141 (1.000)	0.430	3.530 (0.786)	3.321 (0.775)	0.223
Radiomics score ^b^	0.567 (0.495)	0.168 (0.269)	<0.001	0.604 (0.592)	0.164 (0.350)	<0.001	0.627 (0.344)	0.266 (0.281)	0.002

^a^ Data are mean ± standard deviation, ^b^ Data are median (interquartile range)

*GR* Good response, *PR* Poor response, *CEA* Carcinoembryonic antigen, *CA125* Carbohydrate antigen 125, *CA199* Carbohydrate antigen 199, *NAC* Neoadjuvant chemotherapy

### Radiomics feature extraction and selection

**Fig. 2:**
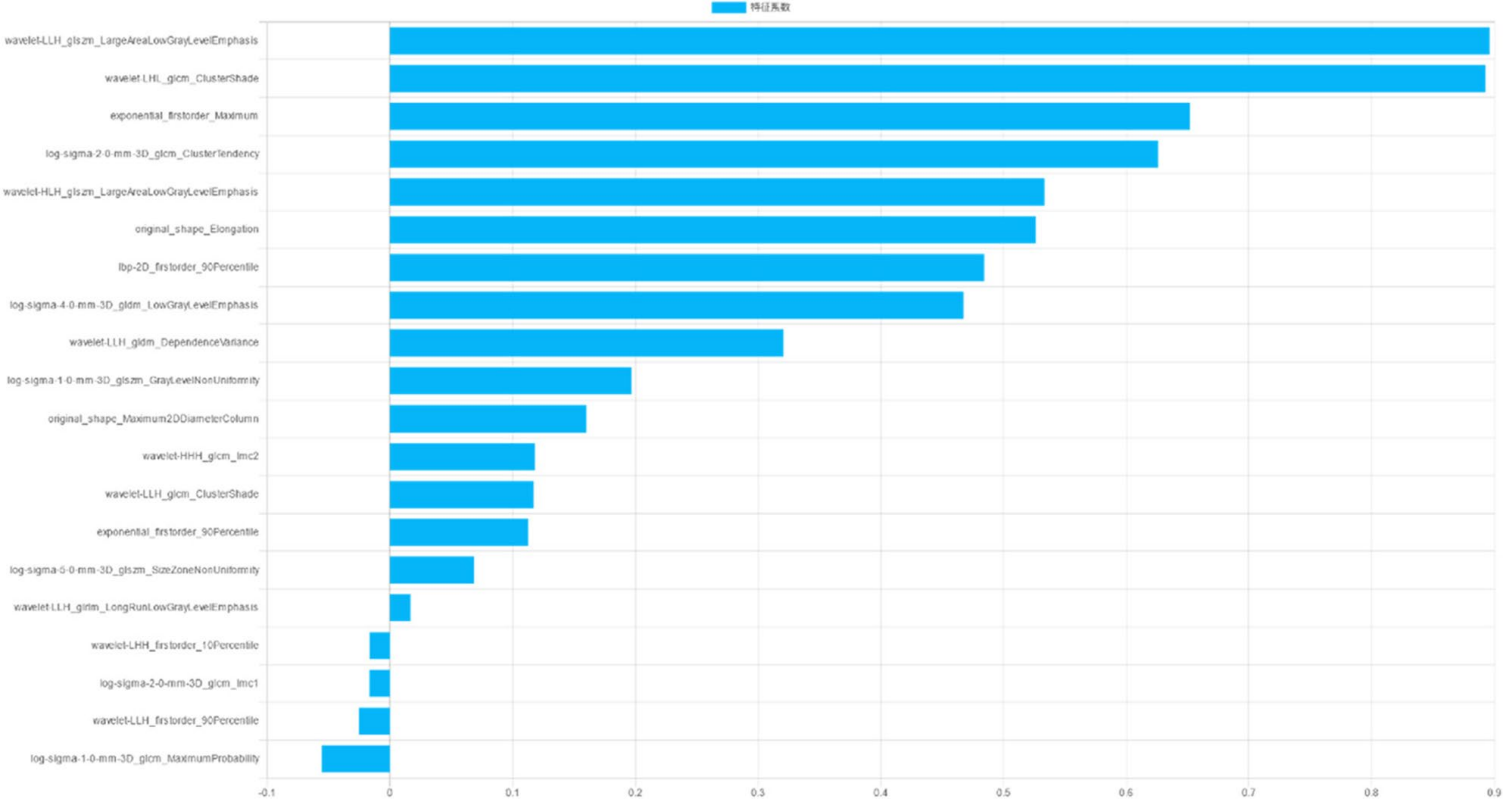
The top twenty feature information and corresponding feature weights of radiomics model in the training cohort

A total of 1874 radiomics features were extracted from the volume of interest of each AEG patient. ICC analysis led to the exclusion of 779 poorly reproducible features (ICC < 0.80). Feature correlation analysis and F-test were performed on the remaining stable radiomics features, and 33 radiomics features were finally retained, as presented in Supplementary Table S1 and Fig. 2. Based on the selected radiomics features, LDA was used as a classifier to construct the radiomics model and calculate the radiomics score.

### Model construction and evaluation

**Fig. 3:**
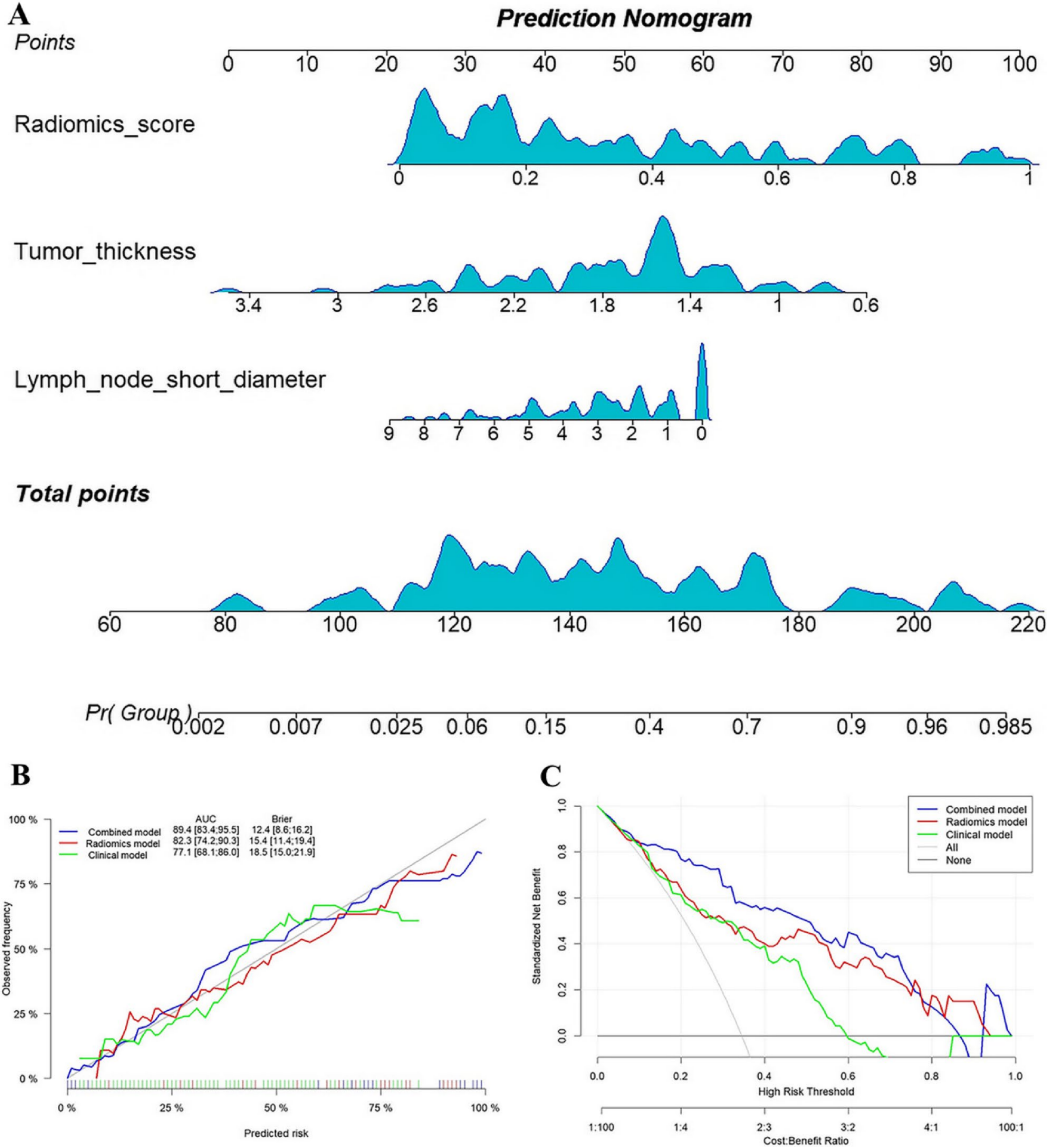
Nomogram developed based on clinical characteristics and radiomics scores and its performance in the training cohort. **A** Nomogram provides a scoring system for predicting response to neoadjuvant chemotherapy for AEG. **B** Calibration curves of radiomics model, clinical model and combined model. **C** Decision curve analysis for radiomics model, clinical model and combined model

**Fig. 4:**
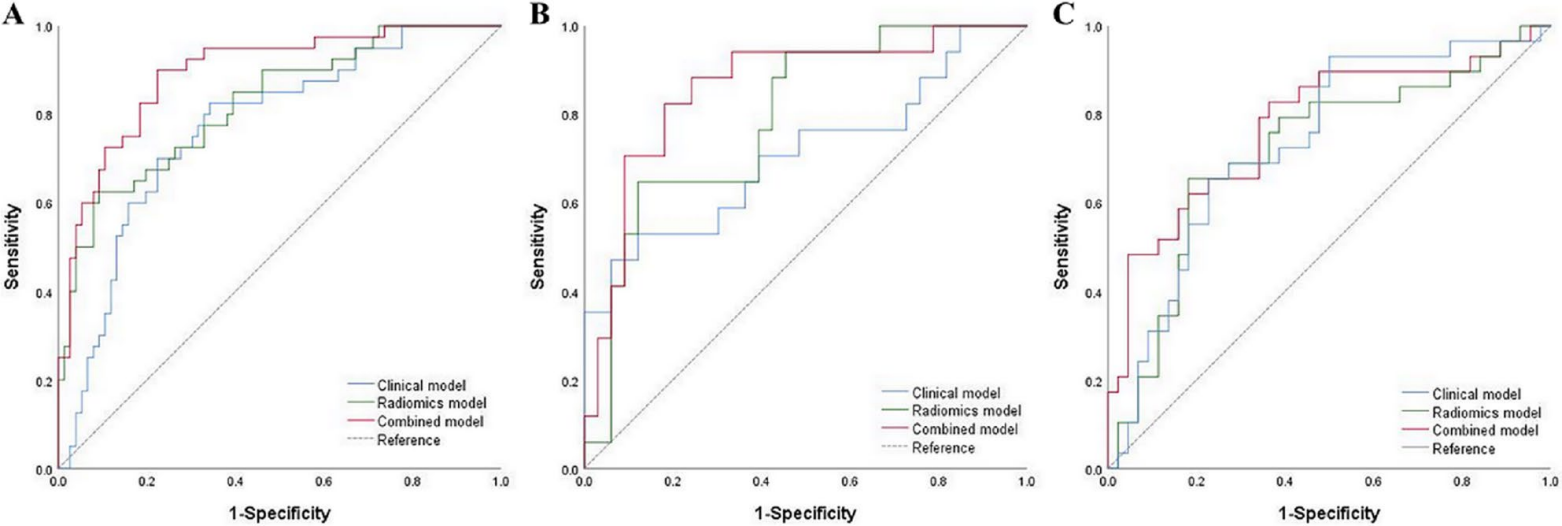
Receiver operating characteristic curves (ROC) for different models in the training, internal verification, and external verification cohorts. **A** The training cohort; **B** The internal verification cohort; **C** The external verification cohort

Multivariable logistic regression was employed to construct a combined model by integrating tumor thickness, lymph node short diameter, and radiomics scores, as listed in Table S2. In the training cohort, the AUC values of the clinical model, radiomics model, and combined model for predicting NAC response were 0.771 (95% CI, 0.682–0.860), 0.823 (95% CI, 0.742–0.903) and 0.894 (95% CI, 0.834–0.954), respectively. Moreover, the DeLong test indicated that the predictive performance of the combined model was superior to that of the clinical and radiomics models (*P* < 0.05), whereas no significant difference was noted in the predictive performance between the clinical model and the radiomics model (*P* = 0.410). The nomogram, calibration curve, and decision curve were generated for the combined model, as depicted in Fig. 3. The calibration and decision curves for the combined model delineated that the model-predicted chemotherapy responses were well-calibrated with actual observations, providing a greater net benefit than the other models. Furthermore, the combined model also exhibited satisfactory predictive performance in the internal and external verification cohorts, with AUC values of 0.859 and 0.775, respectively, as detailed in Table 2; Fig. 4. The calibration curves for the three cohorts unveiled that the predicted values were in good agreement with the actual observed values, and the Hosmer-Lemeshow test showed a good fit for all models (*P* > 0.05). Finally, the decision curves for the two verification cohorts corroborated the clinical utility of the combined model, as shown in Figure S2.

**Table 2:** Comparative performance of clinical, radiomics, and combined models in training and verification cohorts

Cohort	Model	AUC	95%CI	Accuracy	Sensitivity	Specificity
Training	Clinical model	0.771	0.682–0.860	0.733	0.825	0.658
	Radiomics model	0.823	0.742–0.903	0.810	0.625	0.908
	Combined model	0.894	0.834–0.954	0.828	0.900	0.776
Internal verification	Clinical model	0.709	0.545–0.874	0.780	0.471	0.939
	Radiomics model	0.791	0.663–0.920	0.760	0.647	0.879
	Combined model	0.859	0.746–0.972	0.820	0.824	0.818
External verification	Clinical model	0.735	0.618–0.852	0.699	0.931	0.500
	Radiomics model	0.717	0.592–0.842	0.699	0.655	0.818
	Combined model	0.775	0.660–0.890	0.712	0.828	0.636

95%CI, 95% Confidence interval

## Discussion

In recent years, numerous studies have established that patients with gastroesophageal cancer can achieve superior outcomes with neoadjuvant therapy. However, a considerable number of patients do not benefit from the treatment but instead experience treatment-related side effects, with a minority of patients progressing to Stage IV during the treatment course of treatment and are no longer eligible for radical resection [20, 21]. A large clinical study from Europe comparing the efficacy of NAC versus surgery alone in patients with gastroesophageal cancer reported the positive effect of neoadjuvant therapy, with the greatest benefit observed in AEG patients [22]. Notwithstanding, due to its unique anatomical location, several studies have not distinguished AEG from esophageal cancer and gastric cancer, and studies specifically targeting AEG are relatively limited. Herein, a combined model for predicting response to neoadjuvant therapy in AEG patients was developed based on 3D contrast-enhanced CT radiomics features and clinical characteristics and subsequently validated. The results of the study identified radiomics scores, lymph node short diameter, and tumor thickness as independent factors in differentiating the PR and GR groups. As anticipated, the combined model showed excellent predictive performance across all cohorts and may potentially assist in individualized treatment decision-making for AEG patients.

At present, while histopathological examination of surgically resected specimens is the gold standard for evaluating the response to NAC for AEG patients, it is limited by a time lag that impedes early detection of changes in tumor therapy and real-time monitoring of treatment effectiveness. Tumor heterogeneity is an integral factor contributing to the failure of neoadjuvant therapy in cancer patients that can be characterized by radiomics features [23–25]. Cui et al. [26] extracted deep learning and radiomics features from the venous phase CT images of patients with locally advanced gastric cancer and established a combined model. They found that the model exhibited superior performance in predicting the response to NAC, with AUCs of 0.829, 0.804, and 0.827 for the internal and two external validation cohorts, respectively. However, radiomics studies specifically investigating the response to NAC for AEG are rare. Huang et al. [27] constructed radiomics models by extracting two-dimensional radiomics features from the maximal level of tumors of 98 patients with advanced AEG to predict pathological complete response after NAC and documented that the AUC values obtained using venous-phase data were higher than those obtained using arterial-phase data (training: 0.751 vs. 0,736; validation: 0.768 vs. 0,750). Herein, 239 patients with locally advanced AEG from two independent medical centers were divided into the GR group and the PR group. Three-dimensional radiomics features were then extracted from venous phase CT images. Noteworthily, the AUCs of the radiomics model for predicting NAC response were 0.823, 0.791, and 0.717 in the training cohort, internal verification cohort, and external verification cohort, respectively. Figure 2 illustrates the contribution of radiomic features in the model, with large area low gray level emphasis demonstrating the highest impact. This gray-level size zone matrix parameter identifies large, uniform low-attenuation regions, which are strongly indicative of extensive intratumoral necrosis and aggressive tumor behavior. Furthermore, this feature has been leveraged to predict tumor-infiltrating lymphocyte enrichment and is significantly correlated with better outcomes on immune checkpoint inhibitors in non-small cell lung cancer, highlighting its promise as a therapy response biomarker [28].

In addition, the differential diagnostic value of clinical characteristics was explored, identifying lymph node short diameter and tumor thickness as independent influencing factors for predicting the response to NAC for AEG. A previous study identified five risk factors significantly associated with response to NAC in patients with advanced gastric cancer, namely smoking history, clinical T stage, clinical N stage, tumor location, and differentiation [29]. Furthermore, a recent study demonstrated that the reduction rate in the sum of the short diameters of lymph nodes, measured before and after NAC, is significantly associated with overall survival in patients with locally advanced gastric cancer [30]. A larger baseline aggregate lymph node short diameter often signifies a more aggressive tumor phenotype. This heightened aggressiveness, which is frequently associated with molecular characteristics such as genomic instability, uncontrolled cell proliferation, and anti-apoptotic signaling, may also contribute to chemotherapy resistance [31, 32]. Consistent with prior evidence, our study found statistically significant differences in tumor thickness across all cohorts, reaffirming its value in predicting poor response to neoadjuvant therapy [33]. This may be attributed to the fact that increased thickness is often associated not only with greater tumor burden and advanced T-stage but also with features linked to treatment resistance, such as tumor hypoxia and stromal fibrosis. It is hypothesized that these features may collectively establish a barrier, potentially resulting in compromised drug delivery and suboptimal treatment outcomes.

The integration of radiomics with clinical data has been shown to enhance predictive performance, as evidenced in prior studies [34, 35]. In line with this, our combined model demonstrated superior discriminatory power in the training cohort and maintained consistent predictive accuracy across both internal and external verification cohorts. This model enables the pre-therapeutic identification of AEG patients at high risk of poor response to NAC. Patients identified as likely responders can appropriately receive standard NAC, while those predicted to be non-responders may be considered for alternative strategies such as modified chemotherapy regimens, immunotherapy integration, chemoradiation, or expedited surgical intervention, thereby reducing unnecessary treatment-related morbidity and optimizing the timing of curative resection.

This study has several limitations. First, despite being a two-center study, its retrospective nature inherently limits the strength of evidence and may introduce selection bias. Therefore, our findings require validation in larger, prospective, multi-institutional cohorts. Second, although the distribution of NAC regimens did not differ significantly between groups, our study lacked the statistical power to perform a robust, regimen-specific analysis due to the limited sample size. Therefore, their potential confounding effect cannot be ruled out. Finally, the radiomics features were derived from CT images acquired with different scanners and protocols, which may affect feature stability and model generalizability, despite our efforts at image resampling and normalization.

## Conclusion

In summary, this study developed and validated a combined model integrating 3D contrast-enhanced CT radiomics features with clinical characteristics for predicting treatment response to NAC in AEG patients. The model demonstrated good predictive performance and shows potential as a decision-support tool to aid in individualized treatment planning. Prospective validation in multi-center cohorts is warranted to confirm its clinical utility.

## Supplementary Information

Supplementary Material 1.

## Data Availability

Due to privacy regulations, the raw data cannot be made publicly available; however, the datasets utilized and/or analyzed in the current work are available upon reasonable request from the corresponding author ( [Jianbogao0807@163.com](mailto: Jianbogao0807@163.com) ).

## Abbreviations

AEG: Adenocarcinoma of the esophagogastric junction
NAC: Neoadjuvant chemotherapy
CEA: Carcinoembryonic antigen
CA: Carbohydrate antigen
GR: Good response
PR: Poor response
ICC: Intraclass correlation coefficient analysis
LDA: Linear discriminant analysis
ROC: Receiver operating characteristic curve
AUC: Area under the curve
DCA: Decision curve analysis
OR: Odds ratio
CI: Confidence interval
