# Supplementary material for: The value of 3D contrast-enhanced CT radiomics in predicting response to neoadjuvant chemotherapy for adenocarcinoma of the esophagogastric junction: a two-center study
Source: BMC Cancer. 2026 Jan 23;26:259. doi: 10.1186/s12885-026-15609-y (PMC12911287; doi:10.1186/s12885-026-15609-y)
Supplement: Supplementary file 1 — Supplementary Material 1. [file 12885_2026_15609_MOESM1_ESM.docx]

**Supplementary A1.** CT Image Acquisition

Patients were instructed to fast for 6-8 h before the CT examination and were intramuscularly injected with 10-20 mg of scopolamine 15-20 minutes before scanning to reduce gastrointestinal motility (Hangzhou Minsheng Pharmaceutical PG Roup Co., Ltd., specification: 10 mg/ml). In addition, patients consumed 800-1000 mL of warm boiled water 5 minutes before scanning to ensure optimal gastric filling. AEG patients underwent contrast-enhanced CT examinations using two types of CT scanners (Discovery CT 750HD; GE Healthcare, Waukesha, WI, USA or Somatom Force; Siemens Healthineers, Erlangen, Germany). The scanning area extended from at least the lower esophagus to the lower edges of both kidneys. The scanning parameters were as follows: tube voltage 120 kV, tube current 220-330 mA or automatic mA technique, field of view 35-50 cm, matrix 512 × 512, rotation time 0.5-0.8 s, spacing 1.375 or 1.1 mm, scanning layer thickness 5 mm, and reconstruction layer thickness 1.25 mm. The contrast agent iohexol (Shanghai Bolaike Xinyi Pharmaceutical Co., Ltd.) was injected through the cubital vein using a high-pressure syringe, with an iodine concentration of 370 mg/mL, a flow rate of 3.5 mL/s, and a dose of 1.5 mL/kg. Arterial phase and venous phase scans were conducted at 25-30 seconds and 60 seconds, respectively, following the injection of the contrast agent.

**Supplementary A2.** Tumor segmentation and radiomics feature extraction

Radiologists (W.L.M. and Y.Z.) performed image segmentation without accessing any clinical information of patients. A junior radiologist (W.L.M.) delineated the region of interest (ROI) layer by layer along the edge of the tumor on the axial images, avoiding necrotic areas. The ROIs were subsequently combined to form a volume of interest (VOI), as displayed in Figure S1. A senior radiologist (Y.Z.) reassessed and validated the quality of the segmentation until a consensus was reached. To ensure the reproducibility of radiomic features, another senior radiologist (C.M.D.) independently re-delineated the VOI for 30 randomly selected patients.

In addition to the original images, wavelet, Laplacian of Gaussian (LoG), Local Binary Pattern (LBP) 2D, LBP3D, square, square root, logarithm, exponential, and gradient were applied for image conversion. All images were resampled to a new pixel row spacing of 1.0 mm in three dimensions to mitigate the impact of different scanning protocols or scanning equipment on quantitative radiomic features. Radiomics features, including first-order features, shape features, gray-level co-occurrence matrix (GLCM), gray-level size zone matrix (GLSZM), gray-level run length matrix (GLRLM), gray-level dependence matrix (GLDM), and neighboring gray-tone difference matrix (NGTDM), were calculated and extracted from the VOIs of the original and transformed images, respectively, using the Dr. Wise Multimodal Research Platform.

Figure S1. Representative CT images and corresponding VOI segmentation diagrams in the GR group (A) and PR group (B).


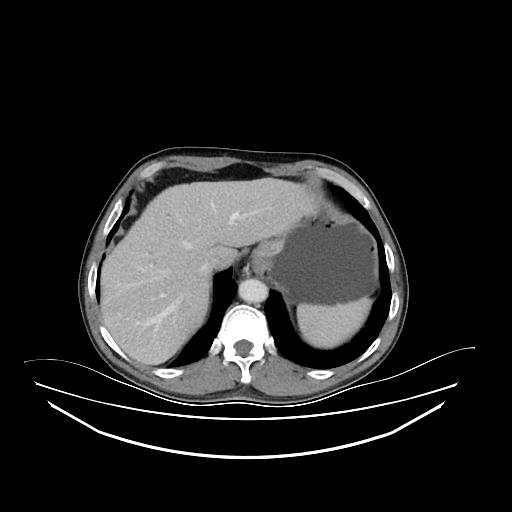

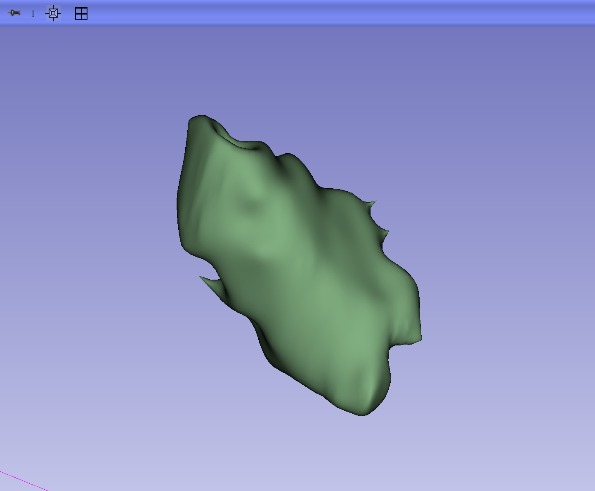

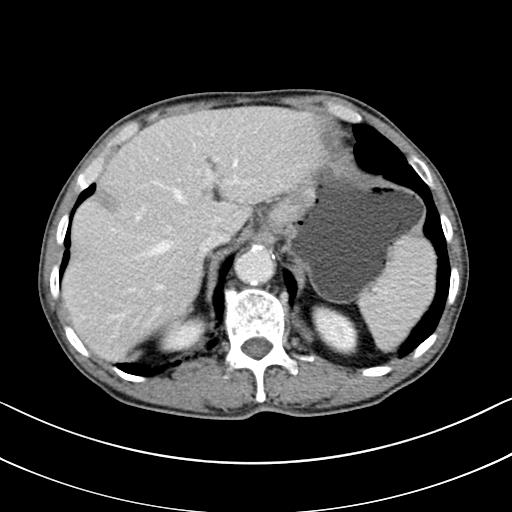

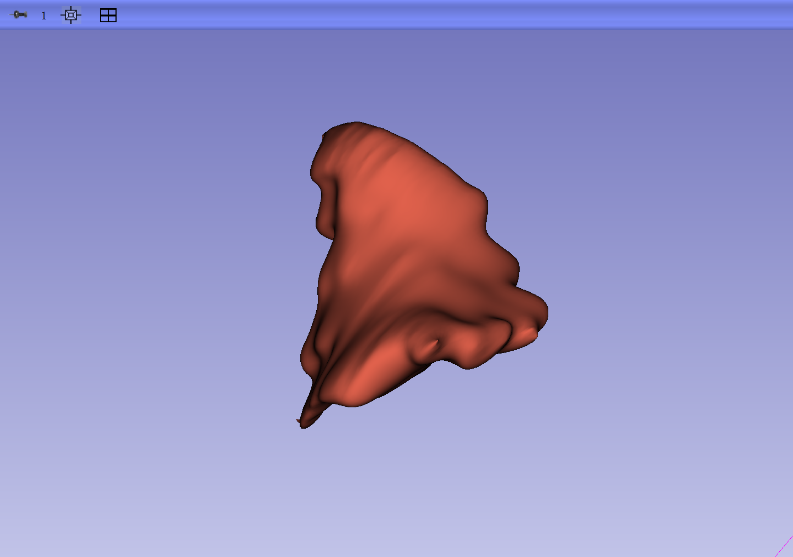


**B**

**A**

**Table S1.** Final retained radiomic features

| **Feature type** | **Feature name** |
| --- | --- |
| shape | original_Elongation |
|  | original_Maximum2DDiameterColumn |
|  | original_Maximum2DDiameterSlice |
|  | original_Sphericity |
| first-order | exponential_90Percentile |
|  | exponential_Maximum |
|  | lbp-2D_90Percentile |
|  | log-sigma-5-0-mm-3D_Skewness |
|  | wavelet-HHH_Skewness |
|  | wavelet-LHH_10Percentile |
|  | wavelet-LHL_Median |
|  | wavelet-LLH_90Percentile |
| glcm | log-sigma-1-0-mm-3D_MaximumProbability |
|  | log-sigma-2-0-mm-3D_ClusterTendency |
|  | log-sigma-2-0-mm-3D_Imc1 |
|  | log-sigma-5-0-mm-3D_ClusterShade |
|  | wavelet-HHH_Imc2 |
|  | wavelet-LHH_MCC |
|  | wavelet-LHL_ClusterShade |
|  | wavelet-LLH_ClusterShade |
|  | wavelet-LLL_InverseVariance |
| gldm | logarithm_DependenceVariance |
|  | logarithm_SmallDependenceHighGrayLevelEmphasis |
|  | log-sigma-4-0-mm-3D_LowGrayLevelEmphasis |
|  | wavelet-LLH_DependenceVariance |
| glrlm | wavelet-LLH_LongRunLowGrayLevelEmphasis |
| glszm | gradient_GrayLevelNonUniformity |
|  | log-sigma-1-0-mm-3D_GrayLevelNonUniformity |
|  | log-sigma-5-0-mm-3D_SizeZoneNonUniformity |
|  | wavelet-HLH_LargeAreaLowGrayLevelEmphasis |
|  | wavelet-LHL_SmallAreaEmphasis |
|  | wavelet-LLH_LargeAreaLowGrayLevelEmphasis |
| ngtdm | gradient_Busyness |

gray-level co-occurrence matrix (glcm), gray-level size zone matrix (glszm), gray-level run length matrix (glrlm), gray-level dependence matrix (gldm), and neighboring gray tone difference matrix (ngtdm)

**Table S2.** Multivariable logistic regression analysis based on the combined model in the training cohort.

| Factor | Regression coefficient | Standard error | P-Value | Odds ratio | 95%CI |
| --- | --- | --- | --- | --- | --- |
| Lymph node short diameter (cm) | -0.324 | 0.148 | 0.029 | 0.724 | 0.542～0.967 |
| Tumor thickness (cm) | -2.059 | 0.642 | 0.001 | 0.128 | 0.036～0.449 |
| Radiomics score (%) | 0.059 | 0.012 | ＜0.001 | 1.061 | 1.035～1.086 |
| Constant | 1.438 | 1.105 | 0.193 | 4.211 |  |

95%CI, 95% Confidence interval


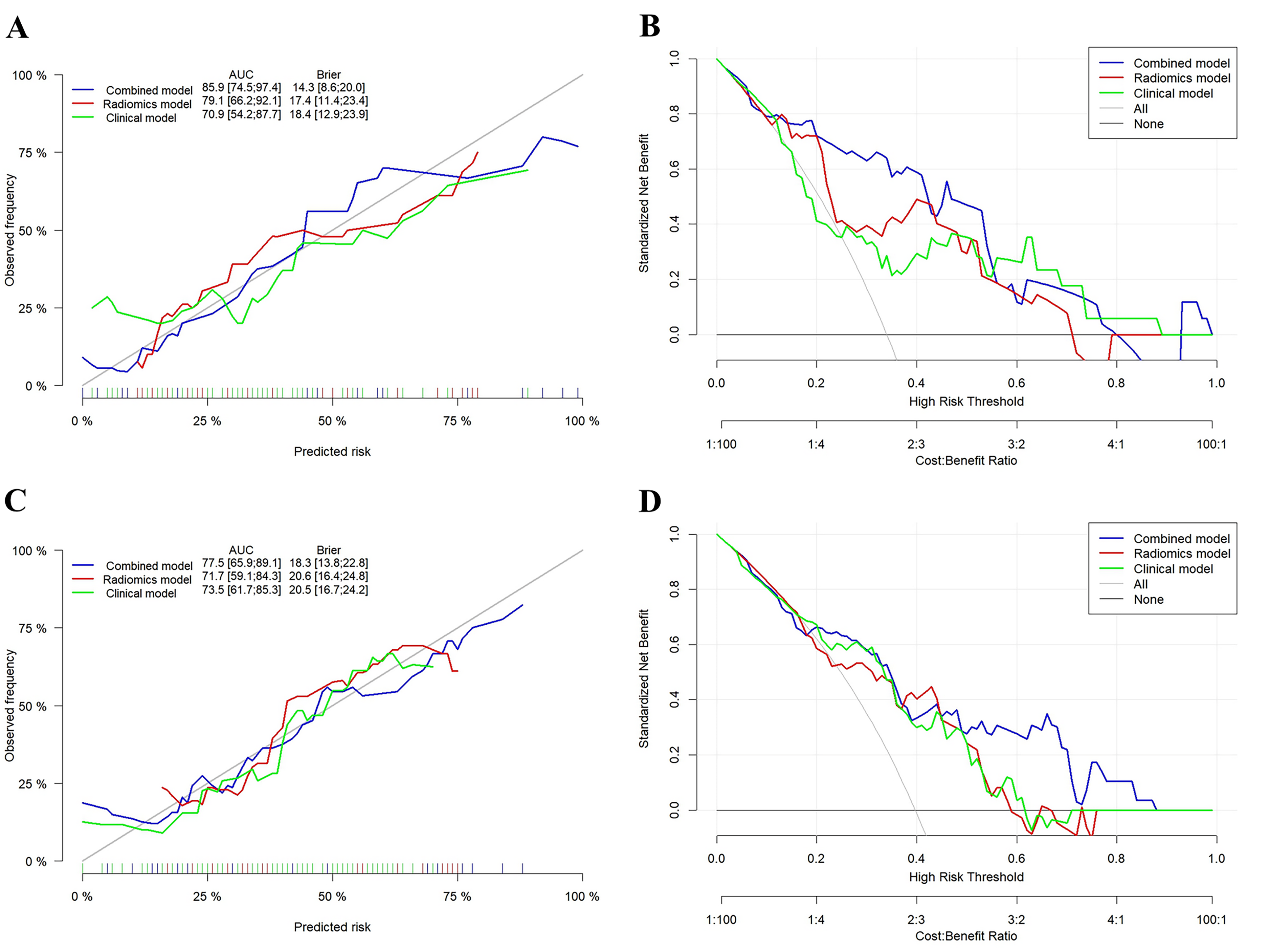


**Figure S2.** Calibration curves and decision curves for internal verification (A, B) and external verification cohort (C, D).
